# When Risk is Unequal: Gendered Pathways to Cardiovascular Disease

**DOI:** 10.5334/gh.1549

**Published:** 2026-04-08

**Authors:** Panniyammakal Jeemon, Susan Uthup, Navya Anikkady

**Affiliations:** 1 Sree Chitra Tirunal Institute for Medical Sciences and Technology, Trivandrum, India

**Keywords:** cardiovascular disease, diabetes, metabolic syndrome, epidemiology

## Abstract

Cardiovascular disease (CVD) continues to represent the leading cause of mortality and poor health worldwide, with low- and middle-income countries (LMICs) experiencing a disproportionate share of the burden. Mexico shows a complex interaction between epidemiological transition, social inequality, and clustered cardiometabolic risk. Data from the 2018 National Health and Nutrition Survey (ENSANUT) in Mexico also revealed gender disparities in cardiovascular risk exposure, symptom perception, health-seeking behavior, treatment patterns, and clinical outcomes. A higher prevalence of obesity, diabetes, and metabolic syndrome, accompanied by delayed diagnosis and underrecognition of cardiovascular risk, was observed in Mexican women. Sociocultural norms, financial dependence, and structural barriers further limit timely access to prevention and care.

Despite advances in healthcare, diagnostic and treatment disparities continue to exist throughout the continuum of cardiovascular care worldwide, contributing to increased mortality. Conventional risk assessment models often fail to account for sex-specific factors, such as reproductive history and lifelong metabolic changes. Reducing these gaps requires redefining cardiovascular risk evaluation, adopting gender-sensitive screening approaches, ensuring equitable access to effective therapies, and implementing policies that address broader social determinants of health. The data from Mexico underscore the importance of developing integrated, gender-responsive cardiovascular care systems capable of lowering preventable deaths while promoting health equity in rapidly evolving societies.

## Cardiovascular Disease at a Crossroads

Cardiovascular disease (CVD) remains the primary global health challenge of the twenty-first century, contributing a major share of annual deaths. It accounts for more than 19.2 million annual deaths, with nearly 80% occurring in low- and middle-income countries (LMICs) (1). Although advances in prevention and treatment have reduced mortality in many high-income settings, these gains have been distributed unevenly. The countries undergoing rapid demographic and nutritional transition encounter an escalation of cardiometabolic risk along with persistent social inequities. These changes have contributed to increased prevalence of obesity, diabetes, and hypertension, resulting in a syndemic characterized by the clustering of multiple chronic conditions within individuals and communities. The National Health and Nutrition Survey 2018 (ENSANUT 2018) suggested that Mexico’s cardiovascular profile has undergone significant shifts in diet, urbanization, and physical activity in recent decades (2).

The paper ‘*Gender Differences in Major Risk Factors for Cardiovascular Disease in Mexican Adults*’ presents a large-scale assessment of CVD risk in Mexico using the data from the ENSANUT 2018 (3). The study highlights that women were more likely to have multiple coexisting risk factors, particularly during midlife (50–59 years), such as a higher burden of obesity, diabetes, dyslipidemia, and hypertension, including evidence linking metabolic risk to social roles, dietary patterns, and lower physical activity (3). Findings from the ENSANUT 2018 offer a comprehensive national overview of these dynamics. It also highlights an underrecognized aspect of gender disparities in CVD care. Cardiovascular risk, outcomes, and the process of care diverge substantially between women and men in Mexico. However, there are limitations to keep in mind when interpreting the results of the CVD assessment in Mexico. The cross-sectional design limits conclusions about causality or the progression of risk factors over time. The study does not fully account for socioeconomic and lifestyle factors such as education, income, diet, smoking, and alcohol use. Some observed sex differences may therefore reflect social and behavioral influences. Greater clarity could be achieved through longitudinal studies and a more detailed classification of CVD outcomes, including sex-specific factors.

## The clustering of risk factors and biological pathways

The cardiometabolic transition in Mexico, as reflected in disease and risk factors, follows the global epidemiological trend. Modernization has altered dietary preferences toward energy-dense processed foods while reducing physical activity in occupations and recreation. It leads to central obesity, which contributes to adipose tissue dysfunction, chronic inflammation, and insulin resistance. These mechanisms accelerate atherosclerosis and intensify the pathogenesis of diabetes and dyslipidemia.

In recent years, cardiovascular risk in Mexico has been driven more by multimorbidity than by single factors. The ENSANUT 2018 data demonstrate exceptionally high levels of metabolic syndrome among Mexican adults, especially women, surpassing international benchmarks (4). Thus, the high burden of abdominal obesity, dyslipidemia, hypertension, and impaired glucose metabolism has become the defining feature of cardiovascular risk in Mexico.

Women carry a disproportionately high burden of risk factors, challenging traditional assumptions of female cardiovascular protection during midlife (5). The co-occurrence of multiple factors, as seen in Mexico, remarkably increases the risk for CVD, particularly among women aged between 50 and 59 years, a critical targeted population for upcoming interventions. The gendered exposures are structured by preferred employment, caregiving responsibilities, and limited opportunities for physical activity, complemented by biological susceptibility. Although the smoking rates are lower among women, the effects are additive due to the differences in the background metabolic environment. Alarmingly, many individuals, particularly women, remain undiagnosed until after a cardiovascular event, revealing missed opportunities for early detection and intervention in primary care. Current screening methods often fail to identify those with risk profiles that differ from the traditional male-based models.

## Gendered inequalities in selected domains

**Figure 1:**
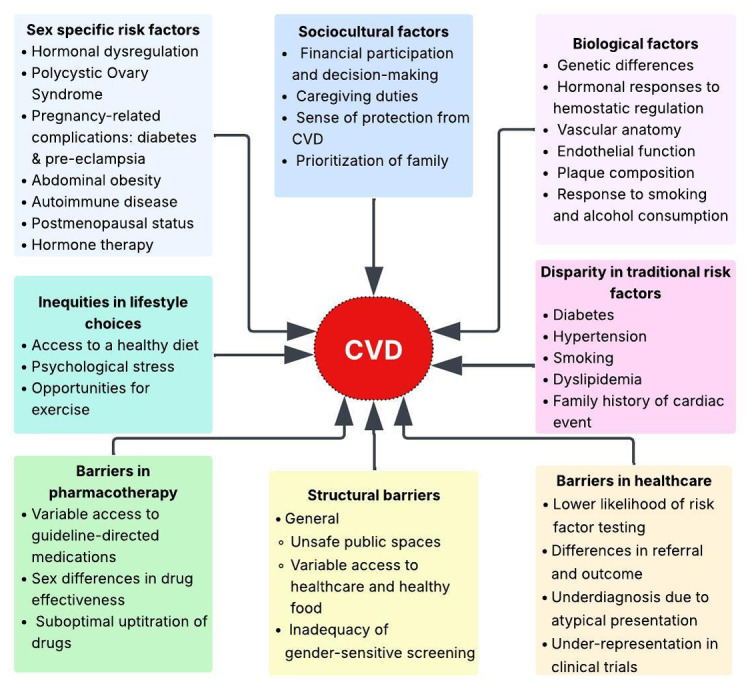
The gendered barriers in cardiovascular disease prevention and care.

Gender differences in CVD are evident in various domains of disease pathogenesis, including clinical presentation, risk perception, social determinants, barriers to care, diagnostic and treatment acquisition, and preventive interventions (Figure 1). In addition to the inherent biological differences, gender disparities are a byproduct of an interconnected pathway spanning physiology, social frameworks, healthcare delivery, and cultural perspectives. Understanding this pathway (Figure 1) is essential for designing prevention strategies to address the escalating cardiovascular burden.

Women experience vague symptoms such as fatigue, nausea, or breathlessness instead of the typical chest pain. These atypical presentations lead to misinterpretation, often delaying evaluation and treatment (6). The perception of heart disease as a disease of men has given a false sense of protection to women, which invariably delays seeking care and reduces preventive initiatives (7). Despite the rising disease burden, the deep-rooted cultural understanding fostered by public health education has reinforced this misconception. The clinical phenotypes of women differ from the classical presentation, which includes heart failure with preserved ejection fraction, valvular disease, and non-obstructive coronary syndromes (8). The varied clinical presentations of women have posed a challenge in early diagnosis, as the diagnostic pathways are tailored to fit the common male presentation of ischemic diseases.

Cardiovascular well-being cannot be separated from the social context in which a person lives. Structural determinants affect a woman’s ability to access preventive and therapeutic services (9). Financial independence and employment in the informal sector reduce access to health insurance and continued care. Thus, the high out-of-pocket costs compel women to prioritize immediate economic needs over long-term treatment. Traditional roles are deeply ingrained in women’s family lives, including caregiving and domestic responsibilities. The caregiving duties further constrain women’s health-seeking behavior. Thus, they prioritize socially expected duties and neglect their own health needs, such as exercise, medical appointments, and participation in rehabilitation (10). Unsafe public spaces indirectly contribute to sedentary lifestyles and unhealthy eating patterns. These intersecting barriers are embedded within the socio-economic structure. Furthermore, pregnancy complications like gestational diabetes mellitus and preeclampsia, early menopause, and autoimmune diseases are sex-specific conditions, which are insufficiently incorporated in routine CVD assessments. Menopause introduces metabolic changes that accelerate vascular disease, while autoimmune conditions disproportionately affecting women further increase risk (11).

The imbalance is evident in the healthcare system as well. Evidence indicates that timely reperfusion during acute myocardial infarction, referral for an invasive intervention, and cardiac rehabilitation are less likely in a female with CVD (12). Such differences cannot be explained solely by clinical presentation; factors such as atypical presentation, which can lead to delayed diagnosis, implicit biases, and logistical barriers, also play a role. The therapeutic effectiveness depends invariably on multiple factors. Adjuvant lifestyle modifications, including targets for body weight, lipid control, and blood pressure management, often represent critical determinants of optimal clinical outcomes. These measures play an essential role in reducing the residual risk that remains despite cardiovascular preventive interventions. However, marked gender disparities persist in the definition, prioritization, and attainment of lifestyle goals (7,8). Social expectations, cultural norms, and differing levels of support frequently influence how targets are set and adhered to, with women often facing structural and societal barriers that limit effective risk reduction within prevailing cultural contexts. The continuum of care is another area where disparities dominate. Access to timely follow-up, structured rehabilitation, or continued lifestyle counseling is limited for women due to a lack of social support, polypharmacy, and transportation barriers (9). These fragmented disparities in delay in diagnosis, treatment inequalities, and structural barriers have cumulative effects on the measurable outcomes.

## Bridging the Gender Divide: Strategic Priorities

A coordinated response that addresses the cardiovascular challenge at multiple levels (Figure 1) is required for effective action. A comprehensive risk assessment tool should be developed that integrates cardiometabolic risk factors such as metabolic syndrome, diabetes, abdominal obesity, and dyslipidemia, while also incorporating sex-specific determinants and the real-world gender disparities that influence access to care, risk recognition, and cardiovascular outcomes. Health system reforms should prioritize equitable access to affordable medications, preventive services, and multidisciplinary cardiovascular care, with specific measures to reduce gender-based barriers to diagnosis and treatment. In parallel, policies that promote healthier food environments and create safe, accessible public spaces for physical activity should be strengthened, recognizing their critical role in addressing gender disparities and supporting effective cardiovascular prevention beyond clinical settings.

Public education campaigns must reframe CVD as a major threat to women’s health, improving symptom recognition and encouraging early care-seeking. Community-based programs and digital health platforms can enhance engagement and continuity. Equally important is reform within clinical research and practice. Greater female representation in trials, routine reporting of sex-disaggregated outcomes, and integration of cardiometabolic therapies with lifestyle interventions are necessary to achieve equitable care.

Regulatory measures, including food labeling, sodium reduction policies, and taxation of unhealthy products, have demonstrated potential to modify population behavior. Gender-responsive health budgeting can ensure equitable allocation of preventive resources. Strengthening primary care systems capable of integrated chronic disease management, with special focus on women’s needs, remains central to sustainable progress.
